# 2-(4-Fluoro­phen­yl)-4-(thio­phen-2-yl)-2,3-di­hydro-1,5-benzothia­zepine

**DOI:** 10.1107/S1600536813025889

**Published:** 2013-10-05

**Authors:** M. Manjula, B. C. Manjunath, N. Renuka, K. Ajay Kumar, N. K. Lokanath

**Affiliations:** a Department of Studies in Physics, Manasagangotri, University of Mysore, Mysore, 570 006, India; b Post Graduate Department of Chemistry, Yuvaraja’s College, University of Mysore, Mysore, 570 006, India

## Abstract

In the title compound, C_19_H_14_FNS_2_, the seven-membered thia­zepine ring adopts a slightly distorted twist boat conformation. The dihedral angle between the benzene rings is 53.6 (1)°. The mean plane of the thia­zepine ring is twisted by 34.3 (7)° and 36.6 (7)° from the benezene rings. A C—H⋯F interaction generates stacking of molecules along the *ab* plane.

## Related literature   

For heterocycles containing the 1,4-thia­zepine ring used as pharmaceutical agents as well as for biologically active compounds, see: Shi *et al.* (2012 ▶). For the pharmacological activity of benzo­thia­zepine and its derivatives, see: Sanjeeva *et al.* (2008 ▶). For standard bond lengths, see: Allen *et al.* (1987 ▶).
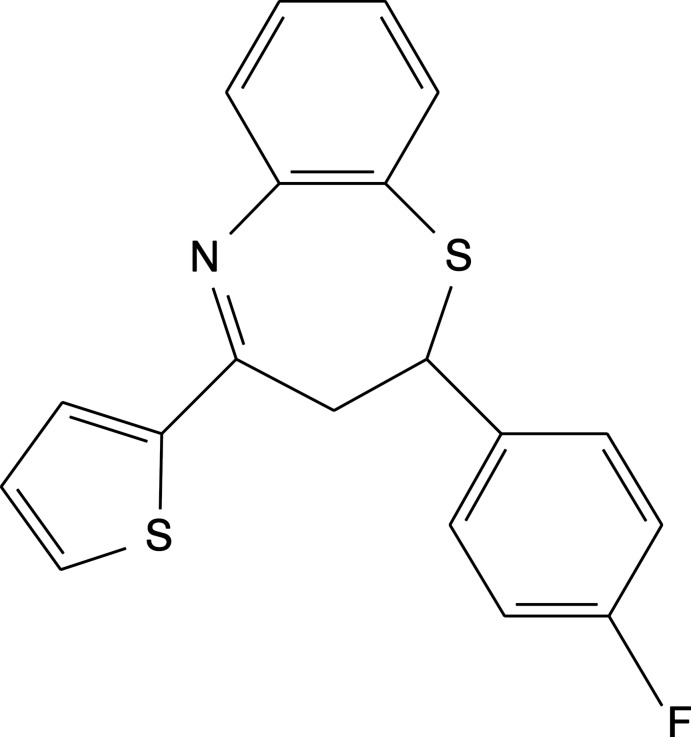


## Experimental   

### 

#### Crystal data  

C_19_H_14_FNS_2_
*M* *_r_* = 339.43
Monoclinic, 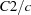

*a* = 26.1463 (17) Å
*b* = 12.3091 (8) Å
*c* = 10.1776 (7) Å
β = 101.383 (4)°
*V* = 3211.1 (4) Å^3^
*Z* = 8
Cu *K*α radiation
μ = 3.07 mm^−1^
*T* = 293 K
0.24 × 0.22 × 0.16 mm

#### Data collection  

Bruker X8 Proteum diffractometer
Absorption correction: multi-scan (*SADABS*; Bruker, 2013 ▶) *T*_min_ = 0.502, *T*_max_ = 0.612
11355 measured reflections
2647 independent reflections
2037 reflections with *I* > 2σ(*I*)
*R*_int_ = 0.054

#### Refinement  

*R*[*F* ^2^ > 2σ(*F* ^2^)] = 0.047
*wR*(*F* ^2^) = 0.134
*S* = 1.05
2647 reflections
209 parameters
H-atom parameters constrained
Δρ_max_ = 0.24 e Å^−3^
Δρ_min_ = −0.29 e Å^−3^

### 

Data collection: *APEX2* (Bruker, 2013 ▶); cell refinement: *SAINT* (Bruker, 2013 ▶); data reduction: *SAINT*; program(s) used to solve structure: *SHELXS97* (Sheldrick, 2008 ▶); program(s) used to refine structure: *SHELXL97* (Sheldrick, 2008 ▶); molecular graphics: *XP* in *SHELXTL* (Sheldrick, 2008 ▶); software used to prepare material for publication: *OLEX2* (Dolomanov *et al.*, 2009 ▶).

## Supplementary Material

Crystal structure: contains datablock(s) I. DOI: 10.1107/S1600536813025889/jj2176sup1.cif

Structure factors: contains datablock(s) I. DOI: 10.1107/S1600536813025889/jj2176Isup2.hkl

Supporting information file. DOI: 10.1107/S1600536813025889/jj2176Isup3.cml

Additional supporting information:  crystallographic information; 3D view; checkCIF report

## Figures and Tables

**Table 1:** Hydrogen-bond geometry (Å, °)

*D*—H⋯*A*	*D*—H	H⋯*A*	*D*⋯*A*	*D*—H⋯*A*
C18—H18⋯F1^i^	0.93	2.72	3.322 (4)	123

Symmetry code: (i) 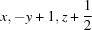.

**Fig. 1.**
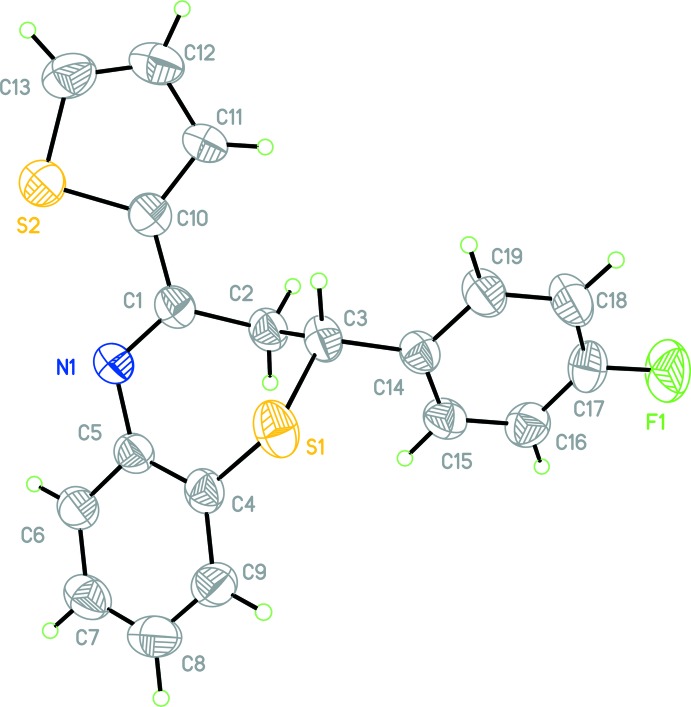
ORTEP diagram of the title molecule, C19H14FNS2, with 50% probability ellipsoids.

**Fig. 2.**
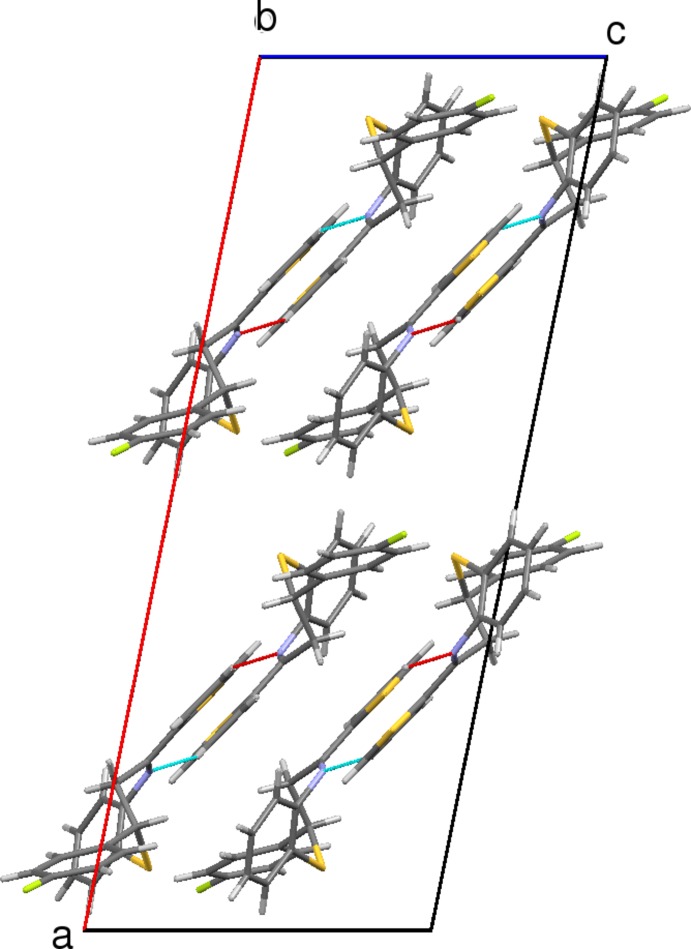
Hydrogen-bond (supramolecular architecure) interactions in the title compound.

## supplementary crystallographic information

### 1. Comment

Heterocycles containing the 1,4-thiazepine ring is one of important moieties in nitrogen and sulfur containing heterocycles and has been widely used as a key building block for pharmaceutical agents as well as for biologically active compounds (Shi *et al.*, 2012). The title compound C_19_H_14_F_N_S_2_, (I), is a tetracyclic structure with one aromatic ring fused to a seven membered ring, on which two heteroatoms are present. Benzothiazepine and its derivatives show a wide spectrum of pharmacological activities such as antifeedent, coronary vasodilatory, tranquilizer, antidepressant, *CNS* stimulant, antihypertensive, calcium channel blocker, antiulcer, calcium antagonist and antimicrobial agents (Sanjeeva *et al.*, 2008). The atoms C9, C8, C11 and C12 present in the central thiazepine ring forms a basal plane with S10 atom as the bow, representing the boat conformation of thiazepine ring.

In (I),The dihedral angle between the mean plans of the benzene rings is 53.6 (1)°. The mean plane of the thiazepine ring is twisted by 34.3 (7)° and 36.6 (7)° from the mean planes of two benezene rings. Bond lengths are in normal ranges (Allen *et al.*,, 1987).

### 2. Experimental

A mixture of 2-aminothiophenol (4 mmol, 0.50 g) with 3-(4-fluorophenyl)-1- (thiophen-2-yl)prop-2-en-1-one (4 mmol, 0.93 g) and 3 to 4 drops of conc. HCl in methanol (10 ml) was heated with stirring at 473 K for 4 h. The reaction was monitored by thin-layer chromatography (hexane or chloroform). After completion of the reaction, the mixture was extracted into ether (30 ml), washed successively with cold and dilute hydrochloric acid and water. The solvent was evaporated to dryness, and crystallized from 95° ethyl alcohol to get pale yellow needles of 2-(4-fluorophenyl)-4-(thiophen-2-yl)-2,3-dihydro-1,5-benzothiazepine in 85° yield. m.p. 422 K. Anal. Calcd. for C_19_H_14_F_N_S_2_: C 67.23, H 4.16, N 4.13°; found C 67.20, H 4.11, N4.08°.

### 3. Refinement

All hydrogen atoms were located geometrically with C—H = 0.93–0.97) Å and allowed to ride on their parent atoms with *U*_iso_(H) = 1.2*U*_eq_(aromatic C).

### Crystal data

**Table:**

C_19_H_14_FNS_2_	*F*(000) = 1408
*M**_r_* = 339.43	*D*_x_ = 1.404 Mg m^−^^3^
Monoclinic, *C*2/*c*	Cu *K*α radiation, λ = 1.54178 Å
*a* = 26.1463 (17) Å	Cell parameters from 11356 reflections
*b* = 12.3091 (8) Å	θ = 64.7–4.0°
*c* = 10.1776 (7) Å	µ = 3.07 mm^−^^1^
β = 101.383 (4)°	*T* = 293 K
*V* = 3211.1 (4) Å^3^	Needle, light yellow
*Z* = 8	0.24 × 0.22 × 0.16 mm

### Data collection

**Table:**

Bruker X8 Proteum diffractometer	2647 independent reflections
Radiation source: Bruker MicroStar microfocus rotating anode	2037 reflections with *I* > 2σ(*I*)
Helios multilayer optics monochromator	*R*_int_ = 0.054
Detector resolution: 10.7 pixels mm^-1^	θ_max_ = 64.7°, θ_min_ = 4.0°
φ and ω scans	*h* = −30→30
Absorption correction: multi-scan (*SADABS*; Bruker, 2013)	*k* = −13→14
*T*_min_ = 0.502, *T*_max_ = 0.612	*l* = −9→11
11355 measured reflections	

### Refinement

**Table:**

Refinement on *F*^2^	Hydrogen site location: inferred from neighbouring sites
Least-squares matrix: full	H-atom parameters constrained
*R*[*F*^2^ > 2σ(*F*^2^)] = 0.047	*w* = 1/[σ^2^(*F*_o_^2^) + (0.071*P*)^2^ + 1.8964*P*] where *P* = (*F*_o_^2^ + 2*F*_c_^2^)/3
*wR*(*F*^2^) = 0.134	(Δ/σ)_max_ < 0.001
*S* = 1.05	Δρ_max_ = 0.24 e Å^−^^3^
2647 reflections	Δρ_min_ = −0.29 e Å^−^^3^
209 parameters	Extinction correction: *SHELXL*, Fc^*^=kFc[1+0.001xFc^2^λ^3^/sin(2θ)]^-1/4^
0 restraints	Extinction coefficient: 0.00022 (8)
Primary atom site location: structure-invariant direct methods	

### Special details

**Table:**

Experimental. ^1^H NMR (CDCl_3_): δ1.82 (d, 1H, C3—H), 2.12 (d, 1H, C3—H), 3.80 (d, 1H, C2—H), 7.20 (dd, 2H, Ar—H), 7.28 (dd, 2H, Ar—H), 7.18 (t, 1H, C4—H 5 m ring), 7.46 (d, 1H, C3—H 5 m ring), 7.72 (d, 1H, C5—H 5 m ring), 7.32–7.46 (m, 4H, Ar—H).
Geometry. All e.s.d.'s (except the e.s.d. in the dihedral angle between two l.s. planes) are estimated using the full covariance matrix. The cell e.s.d.'s are taken into account individually in the estimation of e.s.d.'s in distances, angles and torsion angles; correlations between e.s.d.'s in cell parameters are only used when they are defined by crystal symmetry. An approximate (isotropic) treatment of cell e.s.d.'s is used for estimating e.s.d.'s involving l.s. planes.

### Fractional atomic coordinates and isotropic or equivalent isotropic displacement parameters (Å^2^)

**Table:**

	*x*	*y*	*z*	*U*_iso_*/*U*_eq_	
S1	0.42843 (3)	0.03004 (6)	0.65079 (8)	0.0534 (3)	
S2	0.22949 (3)	−0.03765 (7)	0.72781 (9)	0.0592 (3)	
F1	0.45335 (9)	0.47959 (17)	0.3082 (2)	0.0811 (6)	
N1	0.31778 (8)	−0.09171 (18)	0.5962 (2)	0.0434 (5)	
C1	0.30635 (10)	0.0103 (2)	0.5851 (3)	0.0421 (6)	
C2	0.33154 (10)	0.0903 (2)	0.5056 (3)	0.0446 (7)	
H2A	0.3072	0.1485	0.4743	0.054*	
H2B	0.3398	0.0541	0.4278	0.054*	
C3	0.38127 (10)	0.1381 (2)	0.5894 (3)	0.0434 (6)	
H3	0.3716	0.1717	0.6683	0.052*	
C4	0.40778 (10)	−0.0855 (2)	0.5505 (3)	0.0423 (6)	
C5	0.35838 (10)	−0.1337 (2)	0.5390 (3)	0.0409 (6)	
C6	0.34956 (11)	−0.2345 (2)	0.4772 (3)	0.0471 (7)	
H6	0.3170	−0.2671	0.4692	0.056*	
C7	0.38840 (12)	−0.2870 (3)	0.4272 (3)	0.0543 (8)	
H7	0.3818	−0.3545	0.3862	0.065*	
C8	0.43681 (13)	−0.2397 (3)	0.4380 (3)	0.0588 (8)	
H8	0.4630	−0.2755	0.4053	0.071*	
C9	0.44617 (11)	−0.1395 (3)	0.4974 (3)	0.0515 (8)	
H9	0.4786	−0.1070	0.5024	0.062*	
C10	0.26577 (10)	0.0500 (2)	0.6544 (3)	0.0448 (7)	
C11	0.25218 (11)	0.1577 (2)	0.6726 (3)	0.0499 (7)	
H11	0.2670	0.2175	0.6384	0.060*	
C12	0.21274 (13)	0.1639 (3)	0.7503 (4)	0.0642 (9)	
H12	0.1992	0.2290	0.7748	0.077*	
C13	0.19709 (12)	0.0656 (3)	0.7849 (4)	0.0641 (9)	
H13	0.1713	0.0555	0.8348	0.077*	
C14	0.40372 (10)	0.2271 (2)	0.5150 (3)	0.0406 (6)	
C15	0.41640 (11)	0.2101 (2)	0.3910 (3)	0.0473 (7)	
H15	0.4136	0.1406	0.3544	0.057*	
C16	0.43319 (11)	0.2941 (3)	0.3206 (3)	0.0515 (7)	
H16	0.4410	0.2823	0.2365	0.062*	
C17	0.43805 (12)	0.3947 (3)	0.3772 (3)	0.0526 (7)	
C18	0.42809 (14)	0.4155 (3)	0.5015 (3)	0.0601 (8)	
H18	0.4329	0.4845	0.5391	0.072*	
C19	0.41050 (13)	0.3301 (2)	0.5697 (3)	0.0533 (8)	
H19	0.4031	0.3424	0.6542	0.064*	

### Atomic displacement parameters (Å^2^)

**Table:**

	*U*^11^	*U*^22^	*U*^33^	*U*^12^	*U*^13^	*U*^23^
S1	0.0529 (4)	0.0427 (5)	0.0573 (5)	−0.0034 (3)	−0.0069 (3)	0.0079 (3)
S2	0.0529 (4)	0.0507 (5)	0.0772 (6)	−0.0008 (3)	0.0207 (4)	−0.0009 (4)
F1	0.1102 (16)	0.0648 (13)	0.0754 (14)	−0.0166 (12)	0.0354 (12)	0.0212 (11)
N1	0.0461 (11)	0.0348 (12)	0.0509 (14)	−0.0007 (10)	0.0135 (10)	−0.0027 (11)
C1	0.0417 (13)	0.0372 (15)	0.0453 (16)	−0.0003 (11)	0.0037 (12)	−0.0011 (12)
C2	0.0483 (14)	0.0363 (15)	0.0489 (17)	0.0018 (12)	0.0088 (12)	0.0056 (13)
C3	0.0528 (14)	0.0342 (14)	0.0423 (16)	−0.0060 (12)	0.0079 (12)	−0.0003 (12)
C4	0.0449 (13)	0.0376 (15)	0.0438 (16)	0.0026 (11)	0.0070 (12)	0.0097 (12)
C5	0.0471 (14)	0.0360 (14)	0.0404 (15)	0.0039 (11)	0.0105 (12)	0.0024 (12)
C6	0.0557 (15)	0.0402 (16)	0.0463 (17)	0.0018 (12)	0.0126 (13)	0.0008 (13)
C7	0.0741 (19)	0.0447 (17)	0.0452 (18)	0.0089 (15)	0.0144 (15)	−0.0012 (14)
C8	0.0629 (18)	0.063 (2)	0.054 (2)	0.0211 (16)	0.0197 (15)	0.0065 (16)
C9	0.0466 (14)	0.0549 (19)	0.0554 (19)	0.0096 (13)	0.0159 (13)	0.0145 (15)
C10	0.0413 (13)	0.0436 (16)	0.0481 (17)	0.0001 (11)	0.0049 (12)	−0.0010 (13)
C11	0.0539 (15)	0.0378 (16)	0.0606 (19)	0.0079 (12)	0.0174 (14)	−0.0045 (14)
C12	0.0676 (19)	0.054 (2)	0.073 (2)	0.0165 (16)	0.0189 (17)	−0.0071 (18)
C13	0.0509 (16)	0.073 (2)	0.072 (2)	0.0084 (16)	0.0213 (16)	−0.0012 (18)
C14	0.0483 (14)	0.0358 (14)	0.0369 (15)	0.0002 (11)	0.0067 (11)	0.0007 (12)
C15	0.0564 (15)	0.0418 (16)	0.0452 (17)	−0.0005 (13)	0.0139 (13)	−0.0077 (13)
C16	0.0557 (16)	0.057 (2)	0.0441 (17)	−0.0006 (14)	0.0145 (13)	0.0015 (15)
C17	0.0604 (16)	0.0483 (17)	0.0502 (18)	−0.0065 (14)	0.0138 (14)	0.0156 (15)
C18	0.089 (2)	0.0377 (17)	0.055 (2)	−0.0076 (16)	0.0177 (17)	−0.0019 (14)
C19	0.082 (2)	0.0397 (17)	0.0418 (16)	−0.0034 (14)	0.0202 (15)	−0.0008 (13)

### Geometric parameters (Å, º)

**Table:**

S1—C3	1.837 (3)	C7—C8	1.378 (5)
S1—C4	1.772 (3)	C8—H8	0.9300
S2—C10	1.704 (3)	C8—C9	1.374 (5)
S2—C13	1.693 (3)	C9—H9	0.9300
F1—C17	1.362 (3)	C10—C11	1.394 (4)
N1—C1	1.290 (3)	C11—H11	0.9300
N1—C5	1.406 (3)	C11—C12	1.420 (4)
C1—C2	1.506 (4)	C12—H12	0.9300
C1—C10	1.469 (4)	C12—C13	1.346 (5)
C2—H2A	0.9700	C13—H13	0.9300
C2—H2B	0.9700	C14—C15	1.382 (4)
C2—C3	1.525 (4)	C14—C19	1.382 (4)
C3—H3	0.9800	C15—H15	0.9300
C3—C14	1.515 (4)	C15—C16	1.378 (4)
C4—C5	1.405 (4)	C16—H16	0.9300
C4—C9	1.398 (4)	C16—C17	1.362 (4)
C5—C6	1.389 (4)	C17—C18	1.365 (5)
C6—H6	0.9300	C18—H18	0.9300
C6—C7	1.383 (4)	C18—C19	1.388 (4)
C7—H7	0.9300	C19—H19	0.9300
			
C4—S1—C3	106.11 (12)	C4—C9—H9	119.4
C13—S2—C10	91.98 (16)	C8—C9—C4	121.2 (3)
C1—N1—C5	120.2 (2)	C8—C9—H9	119.4
N1—C1—C2	124.4 (2)	C1—C10—S2	121.1 (2)
N1—C1—C10	117.3 (2)	C11—C10—S2	111.4 (2)
C10—C1—C2	118.3 (2)	C11—C10—C1	127.4 (3)
C1—C2—H2A	109.3	C10—C11—H11	124.5
C1—C2—H2B	109.3	C10—C11—C12	110.9 (3)
C1—C2—C3	111.5 (2)	C12—C11—H11	124.5
H2A—C2—H2B	108.0	C11—C12—H12	123.6
C3—C2—H2A	109.3	C13—C12—C11	112.9 (3)
C3—C2—H2B	109.3	C13—C12—H12	123.6
S1—C3—H3	107.0	S2—C13—H13	123.6
C2—C3—S1	110.59 (18)	C12—C13—S2	112.7 (2)
C2—C3—H3	107.0	C12—C13—H13	123.6
C14—C3—S1	113.15 (18)	C15—C14—C3	122.3 (2)
C14—C3—C2	111.8 (2)	C15—C14—C19	118.1 (3)
C14—C3—H3	107.0	C19—C14—C3	119.6 (2)
C5—C4—S1	123.9 (2)	C14—C15—H15	119.3
C9—C4—S1	116.3 (2)	C16—C15—C14	121.3 (3)
C9—C4—C5	119.0 (3)	C16—C15—H15	119.3
C4—C5—N1	124.6 (2)	C15—C16—H16	120.8
C6—C5—N1	116.4 (2)	C17—C16—C15	118.3 (3)
C6—C5—C4	118.8 (2)	C17—C16—H16	120.8
C5—C6—H6	119.5	F1—C17—C18	117.6 (3)
C7—C6—C5	121.1 (3)	C16—C17—F1	119.5 (3)
C7—C6—H6	119.5	C16—C17—C18	123.0 (3)
C6—C7—H7	119.9	C17—C18—H18	121.2
C8—C7—C6	120.2 (3)	C17—C18—C19	117.6 (3)
C8—C7—H7	119.9	C19—C18—H18	121.2
C7—C8—H8	120.1	C14—C19—C18	121.5 (3)
C9—C8—C7	119.7 (3)	C14—C19—H19	119.3
C9—C8—H8	120.1	C18—C19—H19	119.3
			
S1—C3—C14—C15	69.3 (3)	C4—S1—C3—C2	17.3 (2)
S1—C3—C14—C19	−112.7 (3)	C4—S1—C3—C14	−109.0 (2)
S1—C4—C5—N1	5.5 (4)	C4—C5—C6—C7	−0.1 (4)
S1—C4—C5—C6	−168.7 (2)	C5—N1—C1—C2	4.2 (4)
S1—C4—C9—C8	168.7 (2)	C5—N1—C1—C10	−176.2 (2)
S2—C10—C11—C12	1.5 (3)	C5—C4—C9—C8	−1.8 (4)
F1—C17—C18—C19	177.5 (3)	C5—C6—C7—C8	−0.1 (5)
N1—C1—C2—C3	−87.2 (3)	C6—C7—C8—C9	−0.7 (5)
N1—C1—C10—S2	−8.3 (4)	C7—C8—C9—C4	1.7 (5)
N1—C1—C10—C11	170.2 (3)	C9—C4—C5—N1	175.3 (3)
N1—C5—C6—C7	−174.8 (3)	C9—C4—C5—C6	1.0 (4)
C1—N1—C5—C4	45.9 (4)	C10—S2—C13—C12	0.0 (3)
C1—N1—C5—C6	−139.7 (3)	C10—C1—C2—C3	93.2 (3)
C1—C2—C3—S1	58.9 (3)	C10—C11—C12—C13	−1.5 (4)
C1—C2—C3—C14	−174.0 (2)	C11—C12—C13—S2	0.8 (4)
C1—C10—C11—C12	−177.2 (3)	C13—S2—C10—C1	177.9 (2)
C2—C1—C10—S2	171.3 (2)	C13—S2—C10—C11	−0.9 (2)
C2—C1—C10—C11	−10.1 (4)	C14—C15—C16—C17	1.3 (4)
C2—C3—C14—C15	−56.3 (3)	C15—C14—C19—C18	1.8 (5)
C2—C3—C14—C19	121.6 (3)	C15—C16—C17—F1	−178.4 (3)
C3—S1—C4—C5	−58.1 (3)	C15—C16—C17—C18	1.4 (5)
C3—S1—C4—C9	131.9 (2)	C16—C17—C18—C19	−2.3 (5)
C3—C14—C15—C16	175.2 (3)	C17—C18—C19—C14	0.7 (5)
C3—C14—C19—C18	−176.2 (3)	C19—C14—C15—C16	−2.8 (4)

### Hydrogen-bond geometry (Å, º)

**Table:**

*D*—H···*A*	*D*—H	H···*A*	*D*···*A*	*D*—H···*A*
C18—H18···F1^i^	0.93	2.72	3.322 (4)	123

Symmetry code: (i) *x*, −*y*+1, *z*+1/2.
